# Low frequency of asymptomatic carriage of toxigenic *Clostridium difficile* in an acute care geriatric hospital: prospective cohort study in Switzerland

**DOI:** 10.1186/s13756-016-0123-6

**Published:** 2016-06-08

**Authors:** Daniela Pires, Virginie Prendki, Gesuele Renzi, Carolina Fankhauser, Valerie Sauvan, Benedikt Huttner, Jacques Schrenzel, Stephan Harbarth

**Affiliations:** Infection Control Program, Geneva University Hospitals, 4, rue Gabrielle Perret-Gentil, 1211 Geneva, Switzerland; Department of Infectious Diseases, Centro Hospitalar Lisboa Norte and Faculdade de Medicina da Universidade de Lisboa, Lisbon, Portugal; Department of Geriatrics, Geneva University Hospitals, Geneva, Switzerland; Central Laboratory of Bacteriology, Geneva University Hospitals, Geneva, Switzerland; Department of Infectious Diseases, Geneva University Hospitals, Geneva, Switzerland; Faculty of Medicine, University of Geneva, Geneva, Switzerland

**Keywords:** *Clostridium difficile*, Asymptomatic, Colonization

## Abstract

**Background:**

The role of asymptomatic carriers of toxigenic *Clostridium difficile* (TCD) in nosocomial cross-transmission remains debatable. Moreover, its relevance in the elderly has been sparsely studied.

**Objectives:**

To assess asymptomatic TCD carriage in an acute care geriatric population.

**Methods:**

We performed a prospective cohort study at the 296-bed geriatric hospital of the Geneva University Hospitals. We consecutively recruited all patients admitted to two 15-bed acute-care wards. Patients with *C. difficile* infection (CDI) or diarrhoea at admission were excluded. First bowel movement after admission and every two weeks thereafter were sampled. *C. difficile* toxin B gene was identified using real-time polymerase chain-reaction (BD MAX^TM^Cdiff). Asymptomatic TCD carriage was defined by the presence of the *C. difficile* toxin B gene without diarrhoea.

**Results:**

A total of 102 patients were admitted between March and June 2015. Two patients were excluded. Among the 100 patients included in the study, 63 were hospitalized and 1 had CDI in the previous year, and 36 were exposed to systemic antibiotics within 90 days prior to admission. Overall, 199 stool samples were collected (median 2 *per* patient, IQR 1-3). Asymptomatic TCD carriage was identified in two patients (2 %).

**Conclusions:**

We found a low prevalence of asymptomatic TCD carriage in a geriatric population frequently exposed to antibiotics and healthcare. Our findings suggest that asymptomatic TCD carriage might contribute only marginally to nosocomial TCD cross-transmission in our and similar healthcare settings.

## Introduction

*Clostridium difficile* infection (CDI) is currently the most commonly reported pathogen causing healthcare-associated infections in the United States [1]. Moreover, it is associated with significant morbidity and mortality, particularly among older people [1].

There is ongoing controversy about the role of asymptomatic carriage in the transmission of toxigenic *C. difficile* (TCD) in healthcare facilities [2]. Indeed, asymptomatic TCD carriers have been implicated in TCD cross-transmission in hospitals [3]. Importantly, as they are not a focus of CDI control measures, asymptomatic TCD carriers may constitute an important reservoir for nosocomial transmission [3].

Data on asymptomatic TCD carriage in elderly hospitalized patients remains scarce. In this context, we aimed to assess the prevalence of asymptomatic TCD carriage at admission to an acute care geriatric hospital and the rate of nosocomial acquisition during inpatient stay.

## Methods

The study was performed in the geriatric hospital of the Geneva University Hospitals. In this 296-bed hospital, the incidence of CDI from 1^st^ January until 31^st^ July 2015 was 1.97/10 000 patient-days (without any major clustering). The overall antibiotic use density in 2014 was 220 Defined Daily Doses/1000 patient-days.

All patients consecutively admitted to two 15-bed acute care wards were eligible for inclusion. Patients were recruited from 2^nd^ March until 30^th^ June 2015. Patients with diagnosis of CDI and/or diarrhoea (defined as ≥3 unformed stools/day) during the first 48 h after hospitalization were excluded.

We collected the first bowel movement after admission, and every two weeks thereafter (or earlier, if discharge occurred ≤2 weeks after admission). Patients were followed until discharge. Immediately after a patient’s bowel movement, the stool sample was placed on Cary-Blair® medium for transport to the laboratory. The *C. difficile* toxin B gene was identified using real-time polymerase chain reaction (PCR) (BD MAX^TM^ Cdiff, BD diagnostics, Sparks, Maryland).

Asymptomatic TCD carriage was defined as presence of *C. difficile* toxin B gene without symptoms of diarrhoea at the time of stool collection. If identified in the first 72 h of hospitalization, the case was classified as asymptomatic carrier at admission. If identified after 72 h and with a previous negative result, it was considered as nosocomial acquisition. If the patient was identified after 72 h of hospitalization but without a previous negative sample, we considered it as non-classifiable regarding place of TCD acquisition.

We retrieved information about CDI and hospitalization in the previous year, medications prescribed within 90 days before admission, origin of patient (home, long-term care facility or hospital transfer), demographics, comorbidities, administration of systemic antibiotics during hospitalization and length of stay. Data was manually collected from electronic charts.

CDI patients were placed under contact precautions in single rooms and asymptomatic TCD carriers were submitted to standard precautions. The staff was unaware of a patient’s TCD status. Ethical approval was obtained without the need for individual consent.

## Results

During the study period, 102 patients were admitted to the two acute-care wards. Two patients were excluded from the study at admission (one had CDI and one had diarrhoea). Among the 100 included patients, 63 had been hospitalized and one had had a CDI diagnosis in the previous 12 months. Thirty-six had been exposed to systemic antibiotics, 3 to corticotherapy and 41 to proton pump inhibitors within 90 days before admission. Finally, 7 had been admitted from long-term care facilities (LTCF; Table 1).

**Table 1:** Characteristics of the 100 included patients in the prospective cohort study

Variable	No stool samples *n* = 5	No TCD colonization *n* = 93	TCD asymptomatic carriers *n* = 2*
Male sex	2	21 (23)	1
Age, median years (IQR)	82 (81–83)	85 (80–90)	82/81
Charlson comorbidity index, median (IQR)	8 (5–9)	6 (5–8)	4/6
Medication in the 90 days prior to admission:			
Antibiotic therapy	1	33 (35)	2
Proton pump inhibitors	3	38 (41)	0
Corticotherapy (>25 mg/day)	0	2 (2)	1
Diagnosis of CDI in previous year	0	1 (1)	0
Hospital stay in previous year	4	57 (61)	2
Hospital stay in previous 90 days	3	41 (44)	2
Length of last hospital stay, median days (IQR)	27 (15–37)	16 (5–25)	124/5
Origin at ward admission:			
Home	4	76 (82)	1
LTCF	1	6 (6)	0
Transferred from another ward	0	11 (12)	1
Length of current ward stay in days, median (IQR)	10 (9–14)	24 (14–37)	30/29
Antibiotic therapy during ward stay	2	36 (39)	2

Values are n (%) or as indicated

*Because these data concern only two patients, we decided to specify the values for continuous or categorical variables and to not state *p* values

*CDI* Clostridium difficile infection

*IQR* inter quartile range

*LTCF* long-term care facilities

*TCD* toxigenic Clostridium difficile

A total of 199 stool samples were collected from 95 patients. Twenty-eight patients had only one sample collected, 39 had 2 and 28 had ≥3. We couldn’t obtain samples from five patients because of lack of collaboration on stool collection.

The median number of samples collected *per* patient was 2 [interquartile range (IQR) 1–3]. The first sample was collected at a median of 3 days (IQR, 1–4) and the second sample at a median of 14 days (IQR, 10–17) after admission. Fifty-nine patients had stool samples collected in the first 72 h of hospitalization. For patients for whom two or more samples (67) were collected, the last collection was performed at a median of 6 days (IQR, 2–12) before discharge.

Asymptomatic TCD carriage was identified in two patients. Patient 1 had positive samples at admission (day 1) and at the date of discharge (day 29). Patient 2 had a first test on day 4 that was non-interpretable (due to non-amplification of the internal control of *C. difficile* BD MAX®; this test was not repeated due to lack of remaining sample material) and a second test that was positive on day 17. According to the study definitions, patient 1 was considered asymptomatic TCD carrier at admission and patient 2 was non-classifiable regarding place of TCD acquisition. Both of these patients received antibiotic treatment during hospital stay, and remained asymptomatic. Patient 1 was treated with amoxicillin/clavulanate (2 days) and piperacillin/tazobactam (7 days) for complicated community-acquired pneumonia and patient 2 received ceftriaxone (3 days) and ciprofloxacin (4 days) for community-acquired urinary tract infection. No case of symptomatic CDI was diagnosed within the 2 wards during the study period.

## Discussion

We found a very low frequency of asymptomatic carriage of TCD (2 %) at an acute care geriatric hospital in Switzerland.

This is one of the few studies to address TCD asymptomatic carriage in geriatric population and the first to be conducted in Switzerland. These results provide valuable epidemiological insight into the understanding of CDI, a frequent and severe condition in the geriatric population.

Importantly, our data stand in contrast with similar studies, conducted in heterogeneous acute care hospital populations, that found higher frequencies of asymptomatic TCD carriage. Indeed, frequencies ranging from 4 to 50 % were reported in studies of variable duration (from 2 months up to 1.5 years) and in different settings [2–5]. Our findings are even more striking if we take into account that studies in LTCF and other chronic-care geriatric facilities have also reported higher rates of TCD carriage (up to 51 % during an outbreak in a LTCF) [5, 6].

To explain our results, one might argue that some variables typically associated with asymptomatic TCD carriage, such as history of CDI, corticotherapy or residency in LTCF [4, 5], were not frequent in our population. However, our cohort was mainly constituted by patients with a previous hospitalization and exposed to proton pump inhibitors, and those factors are also frequently linked with TCD carriage [4, 5]. Our patients also had a high rate of antibiotic exposure, both prior to and during hospitalization. However, this variable is considered to be more associated with CDI than with asymptomatic carriage [4, 5].

Our findings and the variable rates of TCD carriage reported in studies from acute care hospitals and LTCF, suggest that factors determining asymptomatic carriage may be multiple, not easily identifiable and still incompletely understood [7].

Recent literature argued for an important contribution of asymptomatic carriers in nosocomial cross-transmission of TCD [3]. On the contrary, our study findings suggest that, in our setting, asymptomatic TCD carriers do not appear to contribute to cross-transmission of TCD. One may hypothesize that, in addition to patient-related risk factors, several healthcare-related aspects may also influence the relative contribution of asymptomatic TCD carriage to the epidemiology of CDI. Those aspects may include the overall CDI incidence (including the occurrence of outbreaks), the pattern of antibiotic utilization and infection prevention practices.

We found four other studies investigating asymptomatic TCD carriage in acute care geriatric hospitals, and only one of them was published in the last 10 years [8–11]. Interestingly, all those studies have found a low prevalence of asymptomatic carriage at admission (0-2 %). Two of them also evaluated the incidence of in-hospital acquisition of asymptomatic TCD carriage, which ranged from 0 to 12 % [8, 10]. The most recent study, performed in 2011 by Schoevaerdts et al*.* during a 1-year period in a 26-bed geriatric hospital, reported a frequency of asymptomatic TCD carriage at admission even lower than ours (2/336), and no cases of in-hospital acquisition of asymptomatic TCD carriage [8]. This study may be criticized since it only included 336 out of 473 (71 %) potentially eligible patients, which could possibly have lead to selection bias [8]. In our study, we included all eligible patients and managed to obtain a very high compliance rate of stool collection (95 %).

Our study has several limitations. First of all, there is no established gold-standard method for the diagnosis of asymptomatic TCD carriage. In our study, we used PCR to identify asymptomatic TCD carriers. Although PCR assays have high sensitivity and specificity and are widely used to diagnose CDI [12], their use to identify asymptomatic TCD carriers has not been established on a definitive base. Concerns may be raised regarding the analytical sensitivity of PCR, because *C. difficile* counts in asymptomatic TCD carriers are lower than in CDI [6]. Donskey et al*.* [13] only identified 68 % of asymptomatic TCD carriers with a commercial PCR assay. The analysis of perirectal swabs of doubtful diagnostic quality and the higher limit of detection of that assay as compared with alternative PCR assays [14–16], may have contributed to that result. Conversely, several large studies used PCR for the diagnosis of asymptomatic TCD carriage and obtained comparable results to studies using toxigenic culture (which is still considered the gold standard for the diagnosis of CDI) [17, 18]. In particular, Hung et al [18] detected more asymptomatic TCD carriers with a PCR assay than with toxigenic culture.

Other limitations worth mentioning are the small sample size of our study and its single center setting. Additionally, it is known that CDI occurrence follows a seasonal pattern, with peaks in winter and early spring [19]. Supposed that TCD carriage follows the same seasonal pattern, the fact that our study was performed from March to June could have slightly underestimated the true carriage rates. Finally, suboptimal compliance with follow-up sampling makes firm conclusions about nosocomial TCD acquisition difficult to establish.

## Conclusions

We found a low prevalence of asymptomatic TCD carriage in geriatric patients, frequently exposed to antibiotics and to the healthcare system. This adds to the ongoing controversy regarding the prevalence and role of asymptomatic TCD colonization in nosocomial cross-transmission. More studies are needed to address *C. difficile* epidemiology in the geriatric population.

## Abbreviations

CDI, *Clostridium difficile* infection; DDD, defined daily doses; IQR, interquartile range; LTCF, long-term care facilities; PCR, polymerase chain reaction; TCD, toxigenic *C. difficile*

## References

[1] Magill SS, Edwards JR, Bamberg W (2014). Multistate point-prevalence survey of health care-associated infections. N Engl J Med.

[2] Donskey CJ, Kundrapu S, Deshpande A (2015). Colonization versus carriage of Clostridium difficile. Infect Dis Clin North Am.

[3] Curry SR, Muto CA, Schlackman JL (2013). Use of multilocus variable number of tandem repeats analysis genotyping to determine the role of asymptomatic carriers in Clostridium difficile transmission. Clin Infect Dis.

[4] Kong LY, Dendukuri N, Schiller I (2015). Predictors of asymptomatic Clostridium difficile colonization on hospital admission. Am J Infect Control.

[5] Furuya-Kanamori L, Marquess J, Yakob L (2015). Asymptomatic Clostridium difficile colonization: epidemiology and clinical implications. BMC Infect Dis.

[6] Riggs MM, Sethi AK, Zabarsky TF, Eckstein EC, Jump RLP, Donskey CJ (2007). Asymptomatic carriers are a potential source for transmission of epidemic and nonepidemic Clostridium difficile strains among long-term care facility residents. Clin Infect Dis.

[7] Harbarth S, Samore MH (2012). Clostridium: transmission difficile?. PLoS Med.

[8] Schoevaerdts D, Swine C, Verroken A, Huang T-D, Glupczynski Y (2011). Asymptomatic colonization by Clostridium difficile in older adults admitted to a geriatric unit: a prospective cohort study. J Am Geriatr Soc.

[9] McCoubrey J, Starr J, Martin H, Poxton IR (2003). Clostridium difficile in a geriatric unit: a prospective epidemiological study employing a novel S-layer typing method. J Med Microbiol.

[10] Rudensky B, Rosner S, Sonnenblick M, Van Dijk Y, Shapira E, Isaacsohn M (1993). The prevalence and nosocomial acquisition of Clostridium difficile in elderly hospitalized patients. Postgrad Med J.

[11] Corrado OJ, Mascie-Taylor BH, Hall MJ, Bolton RP (1990). Prevalence of Clostridium difficile on a mixed-function ward for the elderly. J Infect.

[12] Burnham C-AD, Carroll KC (2013). Diagnosis of Clostridium difficile Infection: an ongoing conundrum for clinicians and for clinical laboratories. Clin Microbiol Rev.

[13] Donskey CJ, Sunkesula VCK, Jencson AL (2014). Utility of a commercial PCR assay and a clinical prediction rule for detection of toxigenic clostridium difficile in asymptomatic carriers. J Clin Microbiol.

[14] BD GenOhmTM Cdif Assay. Package insert. BD diagnostics, San Diego, CA. July 2008. https://www.bd.com/resource.aspx?IDX=17953 (accessed February 2016)

[15] BD MAXTM Cdiff Assay. Package insert. BD diagnostics, Sparks, Maryland. April 2013. http://moleculardiagnostics.bd.com/product/max/ (accessed February 2016)

[16] Xpert® C. difficile. Package insert. Cepheid, Sunnyvale, CA. May 2012. http://www.diagnostictechnology.com.au/persistent/catalogue_files/products/xpertcdifficilepi.pdf (accessed February 2016)

[17] Tschudin-Sutter S, Carroll KC, Tamma PD (2015). Impact of toxigenic clostridium difficile colonization on the risk of subsequent C. difficile infection in intensive care unit patients. Infect Control Hosp Epidemiol.

[18] Hung Y-P, Tsai P-J, Hung K-H (2012). Impact of toxigenic Clostridium difficile colonization and infection among hospitalized adults at a District Hospital in Southern Taiwan. PLoS One.

[19] Furuya-Kanamori L, McKenzie SJ, Yakob L (2015). Clostridium difficile infection seasonality: patterns across hemispheres and continents–A systematic review. PLoS One.

